# Dietary Administration of L-Carnitine During the Fattening Period of Early Feed Restricted Lambs Modifies Ruminal Fermentation but Does Not Improve Feed Efficiency

**DOI:** 10.3389/fphys.2022.840065

**Published:** 2022-02-24

**Authors:** Alba Martín, F. Javier Giráldez, Paola Cremonesi, Bianca Castiglioni, Filippo Biscarini, Fabrizio Ceciliani, Nuria Santos, Sonia Andrés

**Affiliations:** ^1^Department of Nutrition and Production of Herbivores, Instituto de Ganadería de Montaña, CSIC-Universidad de León, León, Spain; ^2^Institute of Agricultural Biology and Biotechnology, Italian National Research Council, Lodi, Italy; ^3^Department of Veterinary Medicine, Università degli Studi di Milano, Milano, Italy

**Keywords:** feed efficiency, lamb, residual feed intake, L-Carnitine, ruminal fermentation, microbiota, feed restriction, nutritional programming

## Abstract

Early feed restriction of lambs may program animals to achieve reduced feed efficiency traits as a consequence of permanent mitochondrial dysfunction. The hypothesis at the background of the present study is that dietary administration of L-Carnitine (a compound that promotes the activation and transportation of fatty acids into the mitochondria) during the fattening period of early feed restricted lambs can: (a) improve the biochemical profile of early feed restricted lambs, (b) improve feed efficiency, (c) modulate the ruminal and intestinal microbiota, and (d) induce changes in the gastrointestinal mucosa, including the immune status. Twenty-two newborn male Merino lambs were raised under natural conditions but separated from the dams for 9 h daily to allow feed restriction during the suckling period. At weaning, lambs were assigned to a control group being fed *ad libitum* a complete pelleted diet during the fattening phase (CTRL, *n* = 11), whereas the second group (CARN, *n* = 11) received the same diet supplemented with 3 g of L-Carnitine/kg diet. The results revealed that even though L-Carnitine was absorbed, feed efficiency was not modified by dietary L-Carnitine during the fattening period (residual feed intake, *p* > 0.05), whereas ruminal fermentation was improved [total short-chain fatty acids (SCFAs), 113 vs. 154 mmol/l; *p* = 0.036]. Moreover, a trend toward increased concentration of butyrate in the ileal content (0.568 vs. 1.194 mmol/100 ml SCFA; *p* = 0.074) was observed. Other effects, such as reduced heart weight, lower levels of markers related to muscle metabolism or damage, improved renal function, and increased ureagenesis, were detected in the CARN group. Limited changes in the microbiota were also detected. These findings suggest that L-Carnitine may improve ruminal fermentation parameters and maintain both the balance of gut microbiota and the health of the animals. However, the improved ruminal fermentation and the consequent greater accumulation of intramuscular fat might have hidden the effects caused by the ability of dietary L-Carnitine to increase fatty acid oxidation at the mitochondrial level. This would explain the lack of effects of L-Carnitine supplementation on feed efficiency and points toward the need of testing lower doses, probably in the context of animals being fed in excess non-protein nitrogen.

## Introduction

The developed countries are implementing approaches for more efficient use of resources by the livestock sector, trying to reduce the “feed conversion rate” (FCR: the amount of feed needed to produce one unit of animal product) by improving feed efficiency of the animals under intensive production systems. It is well known that the efficiency of converting nutrients into gain in sheep is partly under genetic control. Still, other factors, such as health status, digestibility, body protein turnover, gain composition, or the rumen’s microbial community, can also play a role (Cantalapiedra-Hijar et al., 2018; Santos et al., 2018a,b).

Interestingly, many of these factors can be influenced by early nutritional events, giving rise to a new concept called nutritional, metabolic, or developmental programming, which may affect feed efficiency traits during the whole life of the animals (Chavatte-Palmer et al., 2018). Thus, some circumstances during the early life that reduce milk or milk replacer intake (e.g., deficient management practices, reduction of milk production of the dam, pathologies and pain in the udder) cause early feed restriction of the lambs, thus impairing health status and feed efficiency during post-weaning phases, as recently demonstrated in our previous studies (Santos et al., 2018a,b). Accordingly, the higher catabolism of proteins, together with a specific mitochondrial dysfunction causing hypertrophic cardiomyopathy of the heart and increased fat accumulation provoked by a reduction in β-oxidation of fatty acids were identified as likely mechanisms involved in the long-term effects caused by early feed restriction in suckling lambs (Santos et al., 2018b).

Some nutritional approaches to alleviate the adverse effects of early feed restriction (e.g., reduced feed efficiency during the fattening phase) can be suggested based on the knowledge of the underlying mechanisms impairing feed efficiency. The activation and transportation of fatty acids into the mitochondria is a carnitine-mediated entry process that constitutes a rate-limiting factor for fatty acid oxidation (Lehner and Quiroga, 2016). Consequently, it can be hypothesized that dietary administration of L-Carnitine might help to alleviate, at least partially, the reduced feed efficiency of early feed restricted lambs. Although several papers have demonstrated the ability of L-Carnitine to increase the average daily gain in cattle and sheep (White et al., 2001, 2002) other studies have shown no effects either under thermo-neutral or heat stress conditions, despite demonstrating that this compound is absorbed by the animal (Hill et al., 1995; Greenwood et al., 2001; Solhjoo et al., 2014; Hajilou et al., 2015; Ringseis et al., 2018). Therefore, the results are contradictory (Ringseis et al., 2018) and, to our knowledge, no information about the potential of L-Carnitine to increase the feed efficiency of animals with mitochondrial dysfunction caused by early feed restriction is available. At the same time, a proper characterization of ruminal microbiota in these animals is required, given that the rumen microbiome may be modulated by the diet offered, thus improving feed efficiency.

Therefore, to progress beyond the state of the art, this study was designed to test the effects of dietary administration of L-Carnitine during the fattening period of early feed restricted lambs. The initial hypothesis is that the negative effects (originated by early feed restriction) on feed efficiency and health during the fattening period of lambs can be alleviated by dietary administration of L-Carnitine.

## Materials and Methods

All handling practices followed the recommendations of the Directive 2010/63/EU of the European Parliament and the Council on the protection of animals used for scientific purposes, and the experimental protocols were approved by the IGM-CSIC Animal Experimentation Committee (protocol number 2019-04).

### Animals and Diets

Twenty-two newborn male Merino lambs, penned individually with their corresponding ewe during the suckling period, were included in this study. Forty-eight hours after lambing, 19 ewes with single (*n* = 6) or twin births (*n* = 13) were selected, whereas ewes with lambs with lower LBW at birth or whose lambs showed health problems were discarded. The lambs were kept two full days with the mothers to allow colostrum intake and then separated daily from the dams from 9:00 to 18:00 h. At 17:00, all the dams were milked and injected with oxytocin to remove alveolar milk to guarantee milk restriction of the lambs during the natural suckling period. All the lambs were weighed at birth and then twice a week. They were weaned progressively at 40 days of life, restricting the suckling time to 2 h with free access to a complete pelleted diet (CPD) and alfalfa for 7 days. At weaning, lambs were assigned to two experimental treatments (CTRL and CARN groups, *n* = 11 per experimental group), balanced for lambing type (3 and 8 lambs from single and twin births, respectively, in each group), birth weight (3.99 ± 0.178 kg), weight at the end of weaning (11.7 ± 0.35 kg) and growth rate during the lactation period (163 ± 6.7 g/day). During the fattening period, the lambs were distributed in feedlots on sawdust beds, ensuring a minimum space of 2 m^2^/animal. The first feedlot (control group, CTRL) received a completed pelleted diet (CPD) *ad libitum* and the second feedlot (carnitine group, CARN) was fed the same ration formulated with 6 g of Carniking^®^ (50% L-Carnitine, 35% silica, and 15% water, Lonza) per kg. The animals were weighed once a week during the fattening period, and the individual feed intake was monitored by four control feed intake devices in each feedlot (eight devices in total) provided by Agrolaval S.L. (Gijón, Spain) using radio frequency identification (RFID) ear tags. Fresh drinking water was always available.

Samples of the CPD were collected weekly and analyzed for dry matter (DM, ISO 6496:1999), ash (ISO 5984:2002), crude protein (CP, ISO 5983:2009), amylase-treated neutral detergent fiber [(aNDF), NDF was assayed with a heat-stable amylase and expressed inclusive of residual ash; Ankom Technology Corp., Macedon, NY, United States], acid detergent fiber (ADF was expressed inclusive of residual ash; Ankom Technology Corp., Macedon, NY, United States), and total fat (Acid Hydrolysis Filter Bag Technique using the AnkomHCl Hydrolysis System). The ingredients and chemical composition of the CPD administered are summarized in Table 1.

**Table 1:** Ingredients and chemical composition of the complete pelleted diets (control, CTRL and carnitine, CARN) administered during the fattening period of early feed restricted Assaf lambs.

	CTRL	CARN
*Ingredients, g/kg*		
Barley	413	411
Soybean meal, 47% CP	237	236
Corn	150	149
Barley straw	150	149
^1^Vitamin–mineral supplement	23	23
Molasses	20	20
Sodium bicarbonate	7	7
^2^Carniking^®^	0	6
*Chemical composition, g/kg DM*		
DM, g/kg	893	891
^3^aNDF	223	221
ADF	109	108
CP	184	190
Fat	17.0	15.4
Ash	80.2	79.9

1 *Calcium carbonate (10 g), dicalcium phosphate (4 g), salt (5 g), ammonium chloride (2 g), corrector (2 g)*.

2 *Carniking^®^ is formulated with 50% of L-Carnitine*.

3 *Amylase-treated neutral detergent fiber*.

### Biochemical Profile and Quantification of Plasma Carnitine and acyL-Carnitines

All the animals were blood sampled by jugular venipuncture at 08:30 a.m. at three time-points during the experiment (35, 75, and 120 days of age). Tubes containing no anti-coagulant were allowed to clot in a water bath at 37°C for 30 min and then centrifuged at 3520 × *g* for 16 min at 4°C. Serum obtained was frozen at −80°C until used to measure biochemical parameters [Albumin, Aspartato amino transferase (AST), β-hydroxybutyrate (BHB), Total bilirubin, Ceruloplasmin, Creatine Kinase (CK), Creatinine, Triglycerides, Non Esterified Fatty Acids (NEFA), Total cholesterol, High density Lipoprotein (HDL), Low density Lipoprotein (LDL), Gamma glutamyl transpeptidase (GGT), Glucose, Insulin, Protein, Urea, Ca, Mg, and Zn] using clinical chemistry and turbidimetry analyzer Biosystems BA400 (Biosystems S.A., Barcelona, Spain) according to Giráldez et al. (2021).

In addition, another blood sample was collected into tubes containing lithium heparin when the lambs were 4 months old. These tubes were placed in iced water and centrifuged at 3520 × *g* for 16 min at 5°C. Then, plasma samples were stored at −80°C until used to determine both the SOD activity using the SOD Assay Kit (Sigma-Aldrich, St. Louis, MO, United States) according to the manufacturer’s protocol, or the concentration of carnitine and acyL-Carnitines using ultraperformance liquid chromatography coupled to mass spectrometry (UPLC-MS) analyses. An Acquity UPLC HSS T3 1.8 μm, 2.1 × 100 mm column with a pre-column (VanGuard 2.1 mm × 5 mm, 1.8 μm particle size) was used for the liquid chromatography analysis (LC), which was performed in an Acquity Ultraperformance LC (UPLC^®^) from WATERS (Barcelona, Spain). Data were acquired with the software MassLynx^™^, and the software QuantLynx was used for chromatographic peak integration (WATERS, Manchester, United Kingdom). Carnitines were quantified after the peak integration according to a standard curve which was drawn using C16:0d3-carnitine [m/z 403.3621 for (M + H)+], and this standard was also run with the sample set at concentrations of 5 and 1 μg/ml to assess a factor to correct the variability concerning the same concentrations in the standard curve.

### Animal Performance, Feed Efficiency, and Total Apparent Digestibility

The feed conversion rate (FCR) was calculated as the feed to gain ratio [dividing daily dry matter intake (DMI) per day by the average daily gain (ADG, g/day)]. Residual feed intake (RFI) was calculated as the difference between actual DMI and predicted DMI, which was estimated by multiple linear regression using ADG and mean metabolic body weight (MBW, as BW^0.75^) as predictor variables. Residual weight gain (RWG) was also estimated using the following equation: RWG = ADG_i_ – (β_0_ + β_1_ MBW_i_ + β_2_ DMI_i_ + ε_i_). Residual intake and body weight gain (RIG) was calculated as RFI minus RWG, after normalization. Feed and rectal grab samples were collected in each animal for 9 days from the 50 days of the experimental period to determine total apparent digestibility using acid insoluble ash as an internal marker according to Van Keulen and Young (1977), with modifications (Santos et al., 2018b).

### Post Mortem Parameters, Ruminal and Ileal Sampling, SCFAs, and Ammonia Nitrogen (NH_3_-N) Determination

All lambs were slaughtered after a fattening period of 75 days by exsanguination from the jugular vein, eviscerated, and skinned. Feed was withdrawn 2 h before the slaughter. The weight of the organs and fat depots was registered according to Santos et al. (2018b). Ruminal and ileal content from each lamb was removed, mixed, and sampled within 30 min of slaughter, collected in aseptic flasks, frozen at −80°C, freeze-dried, stored at −20°C, and then used for microbial DNA extraction as explained below. In addition, about 200 g of ruminal contents were strained through two layers of cheesecloth, and the pH was measured immediately. Subsequently, 40 ml of ruminal liquid was dispensed into a falcon tube with 1 ml of 20% sulfuric acid solution to acidify the medium and stop the fermentation. Additionally, 3 g of ileal content was mixed with 6 ml of purified Milli-Q water and then acidified using 0.25 ml of sulfuric acid solution. NH_3_-N concentration in the rumen content was determined by a modified colorimetric method (Wheatherburn, 1967) and SCFAs in ruminal and ileal contents were determined according to Saro et al. (2019).

A 20 cm section of the ileum was collected proximal to ileocecal valve and divided into three portions. One of them was immediately stored at −20°C until analysis of immunoglobulin A (IgA). The other two portions were rinsed three times with sterile phosphate-buffered saline solution to remove the digesta and preserved in different ways for analysis of epimural microbial composition (stored at −80°C during 48 h, then freeze-dried) or histological and immunohistological examination (fixed by immersion in 10% buffered formalin for 1 week).

### Microbiota Characterization of Ruminal Content and Ileal Epimural Community

Ruminal content samples were freeze-dried, and stored at −20°C and then used for microbial DNA extraction using the QIAamp Fast DNA Stool Mini Kit (Qiagen) according to the manufacturer instructions with a minor modification. Ileal samples were also freeze-dried, and then, the luminal part of the freeze-dried ileal mucosa was scraped with a scalpel; subsequent microbial DNA extraction with the PureLink^™^ Microbiome DNA Purification Kit (Invitrogen, Germany) was performed according to manufacturer’s instructions. DNA quality and quantity were assessed using a Nanodrop ND-1000 spectrophotometer (NanoDrop Technologies, Wilmington, DE, United States), and the isolated DNA was stored at −20°C until use.

Bacterial DNA was amplified by targeting the V3–V4 hypervariable regions of the 16S rRNA gene (Caporaso et al., 2011) PCR amplification of each sample was performed in a 25-μl volume. A total of 12.5 μl of KAPA HIFI Master Mix 2× (Kapa Biosystems, Inc., MA, United States) were used. Then, 0.2 μl of each primer (100 μM) was added to 2 μl of genomic DNA (5 ng/μl). Blank controls (no DNA template) were also included. Amplification and library quantification were carried out as described previously (Biscarini et al., 2020).

Demultiplexed paired-end reads from 16S rRNA gene sequencing were first checked for quality using FastQC (Simon Andrews, 2010) for an initial assessment. Forward and reverse paired-end reads were joined into single reads using the C++ program SeqPrep (Jonh, 2011). After joining, reads were filtered for quality based on (i) maximum three consecutive low-quality base calls (Phred <19) allowed; (ii) fraction of consecutive high-quality base calls (Phred >19) in a read over total read length ≥0.75; (iii) no “N”-labeled bases (missing/uncalled) allowed. Reads that did not match all the above criteria were filtered out. All remaining reads were combined in a single FASTA file for the identification and quantification of operational taxonomic units (OTUs). Reads were aligned against the SILVA closed reference sequence collection release 132, with 97% cluster identity (Quast et al., 2013; Yilmaz et al., 2014) applying the CD-HIT clustering algorithm (Li and Godzik, 2006). A predefined taxonomy map of reference sequences to taxonomies was then used for taxonomic identification along the main taxa ranks down to the genus level (domain, phylum, class, order, family, and genus). By counting the abundance of each OTU, the OTU table was created and then grouped at each phylogenetic level. OTUs with total counts lower than 10 in fewer than two samples were filtered out. OTU counts were normalized for uneven sequencing depth by cumulative sum scaling CSS (Paulson et al., 2013). All the above steps, except the FastQC reads quality check, were performed with the Quantitative Insights into Microbial Ecology v. 1.9 (QIIME) open-source bioinformatics pipeline for microbiome analysis (Caporaso et al., 2010). More details on the command lines used to process 16S rRNA gene sequence data can be found in Biscarini et al. (2018).

The ileum and rumen microbiota diversity was assessed within (alpha diversity) and across (beta diversity) samples. All indices (alpha and beta diversity) were estimated from the filtered and normalized OTU table. Besides the number of observed OTUs directly counted from the OTU table, within-sample microbial richness, diversity, and evenness were estimated using the following indices: Chao1 and ACE for richness; Shannon, Simpson, and Fisher alpha for diversity (Fisher et al., 1943; Shannon, 1948; Simpson, 1949; Chao, 1984; Chao and Lee, 1992; Chao and Yang, 1993); Simpson E and Pielou J (Shannon evenness) for evenness (Smith and Wilson, 1996). The across-sample microbiota diversity was quantified by calculating Bray–Curtis dissimilarities (Bray and Curtis, 1957). Between-compartment (rumen vs. ileum) and between group (CARN vs. CTRL, separately for rumen and ileum) Bray–Curtis dissimilarities were evaluated non-parametrically using the permutational analysis of variance (PERMANOVA) with 999 permutations (Anderson, 2001). Details on the calculation of the mentioned alpha and beta diversity indices can be found in Biscarini et al. (2018). The Abundance-based Coverage Estimator (ACE) index and sample-based rarefaction were estimated using Python (Biscarini, 2017) scripts. Plots were generated using the ggplot2 R package (Wickham, 2009). Additional data handling and statistical analysis were performed with the R environment for statistical computing (Foundation for statistical Computing, 2015) and with Microsoft Excel.

### Ileal Morphometrical and Immunological Parameters

Formalin-fixed samples from the ileum were trimmed and processed for paraffin embedding and histologic examination (hematoxylin–eosin staining). Slides were examined with a Leica DM2000 LED microscope and digital pictures were taken at 4× magnification. The thickness of mucosa, submucosa, and *tunica muscularis* and the heights of 40 villi were measured at 10 different sites in each picture using the image processing and analysis software ImageJ v1.6.0_14 (National Institutes of Health, United States). The villis height was defined as the distance between the top of the villus and the crypt transition (Makovicky et al., 2014), whereas the crypt depth was obtained according to the single measurements of crypt areas described by Wilson et al. (2018).

Immunohistochemical analysis of the ileal wall samples was performed according to Frutos et al. (2018). Briefly, cross sections were cut from the ileum wall samples and placed onto poly-L-Lysine coated slides for immunohistochemical labeling of T (CD3 antigen), B (CD20 antigen), and M cells (cytokeratin 18 antigen). Quantification of labeled cells (T and B) was performed under a light microscope with a 40× objective, whereas ileal Peyer patches (iPP) domes to quantify M cells labeling intensity were photographed under a 20× objective. The number of T and B cells was counted in ten random fields within the lamina propria of the ileum, whereas quantification of M cells labeling intensity was performed measuring the % of the positive labeled area in the total area of epithelial cells in iPP domes.

Immunoglobulin A was quantified according to the procedure described by Frutos et al. (2018). Briefly, 2 g of ileal mucosa were collected in a falcon tube with 6 ml of ice-cold phosphate-buffered saline solution supplemented with protease inhibitors (Sigma-Aldrich Corp., Saint Louis, Missouri). The protein concentration of each supernatant was adjusted to 500 μg/ml and the amount of IgA was measured using a Genorise ELISA IgA kit (Genorise Scientific, Devon-Berwyn, Pennsylvania).

### RNA Extraction of Ileum Samples and Real-Time Quantitative Reverse Transcription PCR

RNA was extracted according to Frutos et al. (2018) with modifications. Briefly, total RNA was extracted from 50 to 100 mg of ileum samples (preserved in RNAlater) by using the AS1280 Maxwell^®^ 16 LEV simplyRNA Purification Kit (Promega), according to manufacturer’s guidelines. The RNA quantity was measured using the QuantiFluor^®^ RNA System and the QuantusTM Fluorometer (Promega) and RNA integrity number was determined using Bioanalyzer 2100 (Agilent Technologies, Santa Clara, California). Total RNA was reversed transcribed to cDNA using the Invitrogen^™^ SuperScript^™^ VILO^™^ Master Mix (Thermo Fisher Scientific), according to the manufacturer’s instructions.

In the ileal tissue, the expression of four genes encoding cytokines (IFN-γ, TGF- β, IL-4, IL-10) and four tolls like receptors (TLR-3, TLR-6, TLR-7, TLR-10) was assessed using the gene-specific primer pairs described by Martínez-Pérez et al. (2014) and Frutos et al. (2018). Briefly, for each PCR reaction, 50 ng of cDNA were amplified with each primer set using a Bio-Rad iCycler (Bio-Rad Laboratories Ltd., Mississauga, Ontario) and the amplification conditions described by Frutos et al. (2018). Amplification data were expressed using the 2^–ΔCt^ method (Schmittgen and Livak, 2008), where ΔCt value is the change in quantification cycle calculated as ΔCt = Ct (target gene) – Ct (β-actin). A higher 2^−ΔCt^ value equates to a more abundant transcript. The fold change in expression between CARN and CTRL lambs is the ratio between the average values of 2^–ΔCt^ for the experimental treatments. Fold change due to the treatment is calculated as −1/fold change ratio.

### Statistical Analyses

Data of carnitine and acyL-Carnitines in plasma, animal performance, total apparent digestibility, ruminal and ileal fermentation parameters, organ weight and fat depots, histological, morphological, immunohistochemical data, IgA, and gene expression of ileal samples were analyzed by one-way ANOVA using the GLM procedure of SAS (SAS Institute Inc., Cary, North Carolina) with dietary treatment (CARN vs. CTRL) as the only tested factor. In all cases, the individual lamb was considered as the experimental unit.

Data corresponding to biochemical parameters measured at several growth stages (35, 75, and 120 days of life) were analyzed as a repeated measures design using the MIXED procedure of SAS. Dietary treatment and growth stage were included in the model (the main effects of both factors and their interaction) as fixed effects. In all cases, the individual lamb was considered as the experimental unit and included in the model as a random effect. For each variable, the statistical model was fitted with different competing covariance structures (compound symmetry, unstructured and autoregressive), selected with the smallest value for Akaike’s and Bayesian criteria. The Tukey–Kramer multiple-comparison test was used to compare means when the interaction between dietary treatment and growth stage was significant (value of *p* < 0.05). Value of *p* in-between 0.05 and 0.10 was considered as trend.

## Results

### Biochemical Profile and Quantification of Plasma Carnitine and acyL-Carnitines

According to the biochemical profile data (Table 2), no effects on most of the parameters studied were detected (e.g., NEFA, BHB, cholesterol, glucose, insulin, and total protein in plasma) between groups. The concentration of creatinine decreased in a statistically significant way in the CARN group (0.838 vs. 0.788 mg/dl; *p* = 0.049), whereas creatine kinase trended toward significant increments (219 vs. 297 U/l; *p* = 0.062) when feeding carnitine during the fattening period. No differences were observed in SOD activity during the fattening period (68.8 vs. 69.7% of inhibition rate; *p* = 0.724). Regarding the concentration of plasma carnitine and acyL-Carnitines during the fattening period (Table 3) most of these metabolites were over-accumulated in the CARN group [e.g., carnitine, C4:0(CH_3_) isovaleryl, and C16:0; *p* < 0.01].

**Table 2:** Biochemical parameters of early feed restricted lambs being fed the control (CTRL) or the L-Carnitine diet (CARN) during the fattening period.

	CTRL	CARN^1^	Growth stage			SED^2^	SED^3^	Value of *p*		
			35 days	75 days	120 days			Group	Day	G*Day
Albumin (g/L)	37.2	36.4	37.5^b^	34.9^a^	38.0^b^	0.776	0.668	0.303	<0.001	0.092
Protein (g/L)	57.9	59.2	59.82	57.88	57.94	0.844	0.984	0.126	0.093	0.906
Urea (mg/dl)	38.5	42.5	41.03^b^	34.64^a^	45.83^b^	2.301	2.412	0.099	<0.001	0.102
Creatinine (mg/dl)	0.838	0.788	0.814^b^	0.752^a^	0.872^c^	0.024	0.024	0.049	<0.001	0.041
Creatine kinase (U/L)	468	297	187^a^	325^a^	636^b^	92.8	121.3	0.081	0.002	0.067
Glucose (mg/dl)	89.1	86.1	91.36	80.3	81.16	6.464	6.849	0.655	0.195	0.796
Insulin (uUI/ml)	34.3	31.5	47.47^b^	12.96^a^	41.84^b^	14.04	13.37	0.736	0.023	0.458
BHB^4^ (mmol/L)	7.79	7.31	6.10^a^	6.55^a^	10.00^b^	0.701	0.858	0.505	<0.001	0.990
NEFA^5^ (mmol/L)	0.163	0.195	0.321^b^	0.111^a^	0.105^a^	0.038	0.047	0.421	<0.001	0.123
Triglycerides (mg/dl)	22.0	21.0	33.16^b^	15.74^a^	15.69^a^	3.237	3.722	0.767	<0.001	0.960
Cholesterol (mg/dl)	67.9	63.9	121.4^b^	36.9^a^	39.3^a^	7.550	8.829	0.605	<0.001	0.908
HDL^6^ (mg/dl)	38.6	35.7	60.84^b^	25.14^a^	25.42^a^	2.470	2.970	0.246	<0.001	0.884
LDL^7^ (mg/dl)	21.5	19.5	38.5^b^	10.97^a^	12.09^a^	3.885	4.268	0.621	<0.001	0.894
Total bilirubin (mg/8 L)	0.502	0.578	0.839^c^	0.459^b^	0.324^a^	0.073	0.067	0.307	<0.001	0.499
AST^8^ (U/L)	132	105	60.1^a^	87.1^a^	208.4^b^	21.2	25.7	0.205	<0.001	0.043
GGT^9^ (U/L)	85.7	86.8	81.62^ab^	79.1^a^	97.97^b^	7.248	6.398	0.889	0.010	0.798
Ceruloplasmin (mg/dl)	2.80	3.05	4.09^c^	2.83^b^	1.85^a^	0.315	0.277	0.430	<0.001	0.772
Ca (mg/dl)	10.9	10.9	10.7^a^	10.9^ab^	11.1^b^	0.187	0.142	0.657	0.023	0.327
Mg (mg/dl)	2.54	2.49	2.48	2.49	2.57	0.062	0.056	0.493	0.269	0.539
Zn (μg/dl)	52.0	49.1	49.47	50.75	51.39	4.696	4.300	0.551	0.902	0.239

1 *CARN, lambs received a diet including 3 g of L-Carnitine/kg*.

2 *SED, standard error of the difference to compare experimental groups*.

3 *SED, standard error of the difference to compare days*.

4 *BHB, beta-hydroxybutyrate*.

5 *NEFA, non-esterified fatty acid*.

6 *HDL, high density lipoprotein*.

7 *LDL, low density lipoprotein*.

8 *AST, aspartate aminotransferase*.

9 *GGT, gamma-glutamyl transpeptidase*.

*Different superscripts (a, b) in the same line indicate statistical differences (p < 0.05) between days*.

**Table 3:** Plasma carnitine and acyL-Carnitine concentrations (μg/ml) of early feed restricted lambs being fed the control (CTRL) or the L-Carnitine diet (CARN) during the fattening period.

	CTRL	CARN^1^	SED^2^	Value of *p*
Carnitine	0.035	0.122	0.0052	<0.001
C2:0 (acetyL-Carnitine)	0.017	0.101	0.0113	<0.001
C4:0(CH_3_) isovaleryl	0.027	0.074	0.0112	<0.001
C16:0	0.021	0.029	0.0028	0.007
C18:1	0.006	0.013	0.0042	0.128
C18:0	0.055	0.064	0.0171	0.637
C20:2(OH)	0.025	0.020	0.0029	0.072
C22:0 Taurine/C24:1(OH)2 FA	0.011	0.010	0.0030	0.624

1 *CARN, lambs received a diet including 3 g of L-Carnitine/kg*.

2 *SED, standard error of the difference*.

### Animal Performance, Feed Efficiency, and Digestibility

No differences were observed in animal performance during the suckling period (average daily gain) or in feed efficiency indexes (fattening period), such as residual feed intake (Table 4, RFI = −25.9 vs. 25.9 g/day for CTRL and CARN, respectively; *p* = 0.142). However, a trend toward significantly increased coefficients of dry matter digestibility was detected in the CARN group during the fattening period (Table 4, 48.7 vs. 53.6; *p* = 0.079).

**Table 4:** Pre and post-weaning growth performance, feed efficiency, digestibility, and visceral organ weights (kg) and fat depots (kg) of early feed restricted lambs being fed the control (CTRL) or the L-Carnitine diet (CARN) during the fattening period.

	CTRL	CARN^1^	SED^2^	Value of *p*
*Pre-weaning period*				
Birth weight (kg)	4.00	3.97	0.365	0.939
ADG (g/day)^3^	168	158	13.6	0.435
Weaning weight (kg)^4^	11.93	11.40	0.697	0.452
*Fattening period*				
DMI^5^ (g/day)	993	1,052	45.4	0.205
ADG (g/day)^6^	280	302	17.4	0.216
Feed to gain ratio (g DMI/g ADG)	3.63	3.49	0.212	0.528
Residual Feed Intake	-25.9	25.9	33.95	0.142
Residual Weight Gain	−8.30	8.30	12.690	0.206
Residual intake and body weight gain	−0.042	0.042	0.6805	0.904
*Digestibility (coefficient)*
Dry matter	48.7	53.6	2.68	0.079
Organic matter	52.4	56.8	2.55	0.121
Slaughter weight (kg)	33.4	34.7	1.95	0.521
Full digestive weight (kg)	8.62	9.38	0.648	0.256
Blood (kg)	1.43	1.37	0.094	0.499
Heart (kg)	0.213	0.167	0.0284	0.027
Respiratory tract (kg)^6^	0.610	0.650	0.0562	0.485
Liver (kg)	0.826	0.900	0.0695	0.303
Spleen (kg)	0.104	0.121	0.0170	0.342
Omental fat (kg)	0.323	0.311	0.0525	0.895
Kidney knob and channel fat (kg)	0.231	0.200	0.0323	0.344
Intramuscular fat^7^	2.65	3.36	0.334	0.047
Mesenteric fat (kg)	0.284	0.282	0.0269	0.952

1 *CARN, lambs received a diet including 3 g of L-Carnitine/kg*.

2 *SED, standard error of the difference*.

3 *ADG, Average Daily Gain*.

4 *Weight at the end of the weaning period*.

5 *DMI, dry matter intake*.

6 *pharynx, trachea, lungs*.

7 *Intramuscular fat (% of fresh matter in longissimus dorsi)*.

### Post Mortem Parameters, Ruminal and Ileal Sampling, SCFAs, and Ammonia Nitrogen (NH_3_-N) Determination

The heart weight was significantly reduced in the CARN group (0.213 vs. 0.167 kg; *p* = 0.027; Table 4), whereas the intramuscular fat content was increased (2.65 vs. 3.36% fresh matter of muscle *longissimus dorsi*; *p* = 0.047; Table 4). No significant differences were detected for the rest of the non-carcass fat depots or visceral weights (Table 4).

Additionally, the ruminal pH was lower in the CARN group (Table 5, 5.70 vs. 5.18; *p* = 0.023), whereas the concentration of total SCFA (113 vs. 154 mmol/l; *p* = 0.036) and the proportion of propionate (18.5 vs. 22.6 mmol/100 mmol SCFA; *p* = 0.079) were increased. On the other hand, the ratios of isovalerate (1.50 vs. 0.65 mmol/100 mmol SCFA; *p* = 0.045) and isobutyrate (1.20 vs. 0.67 mmol/100 mmol SCFA; *p* = 0.040) were lower in the rumen of the CARN group. At the ileal level, a trend toward significantly increased proportion of butyrate very close to statistical significance was detected in the CARN group (Table 5; 0.568 vs. 1.194 mmol/100 mmol SCFA; *p* = 0.074), whereas the proportion of acetate trended toward decreased values (Table 5; 97.5 vs. 95.9, *p* = 0.089). Both valerate and isovalerate were significantly reduced in the ileum of the CARN lambs (*p* < 0.05). Ammonia nitrogen concentration was not affected by dietary treatments (*p* > 0.05).

**Table 5:** Ruminal pH and short-chain fatty acids (SCFA) profile at ruminal and ileal level of early feed restricted lambs being fed the control (CTRL) or the L-Carnitine diet (CARN) during the fattening period.

	CTRL	CARN^1^	SED^2^	Value of *p*
*Ruminal pH and fermentation end products*				
pH	5.70	5.18	0.214	0.023
SCFA (mmol/L)	113	154	18.2	0.036
*Molar proportions (mmol/100 mmol SCFA)*
Acetate	55.4	54.6	3.57	0.832
Propionate	18.5	22.6	2.22	0.079
Butyrate	19.8	17.4	3.12	0.444
Valerate	2.73	3.07	0.323	0.305
Caproate	0.92	1.06	0.315	0.651
Isovalerate	1.50	0.65	0.401	0.045
Isobutyrate	1.20	0.67	0.243	0.040
Ammonia nitrogen (mg/L)	95.2	92.4	35.70	0.939
*Ileal SCFA (mmol/l)*	11.7	10.3	0.550	0.014
*Molar proportions (mmol/100 mmol SCFA)*
Acetate	97.5	95.9	0.872	0.089
Propionate	1.44	1.76	0.290	0.289
Butyrate	0.568	1.194	0.331	0.074
Valerate	0.116	0.422	0.142	0.043
Isovalerate	0.331	0.501	0.081	0.049
Isobutyrate	0.034	0.086	0.035	0.148

1 *CARN, lambs received a diet including 3 g of L-Carnitine/kg*.

2 *SED, standard error of the difference*.

### Microbiota Characterization of Ruminal Content and Ileal Epimural Community

The alpha diversity indices measured in the carnitine and control groups from microbiota data in the rumen and ileum compartments are reported in Table 6. The ACE Chao1 and Simpson E indices were significantly different in the ileum of CARN and CTRL lambs, while only the Simpson index was significantly different between groups in the rumen. However, the equitability index in the ileum, and the ACE, Chao1, Shannon observed OTUs indices in the rumen were very close to the significance threshold (Table 6).

**Table 6:** Alpha diversity indices estimated from the rumen and ileum microbiota composition of early feed restricted lambs being fed the control (CTRL) or the L-Carnitine diet (CARN) during the fattening period.

Type	*N*	Metric	CTRL	CARN^1^	Value of *p*
Ruminal	11	ace	1180.614	1004.145	0.066
Ruminal	11	chao1	1201.776	994.114	0.052
Ruminal	11	equitability	0.957	0.958	0.893
Ruminal	11	fisher_alpha	459.337	401.554	0.091
Ruminal	11	observed_otus	875.818	771.636	0.051
Ruminal	11	shannon	9.348	9.169	0.055
Ruminal	11	simpson	0.994	0.998	0.047
Ruminal	11	simpson_e	0.594	0.598	0.768
Ileal	11	ace	937.415	1147.411	0.027
Ileal	11	chao1	917.838	1180.077	0.009
Ileal	11	equitability	0.963	0.959	0.061
Ileal	11	fisher_alpha	423.171	466.812	0.313
Ileal	11	observed_otus	762.727	829.182	0.313
Ileal	11	shannon	9.199	9.274	0.573
Ileal	11	simpson	0.998	0.998	0.748
Ileal	11	simpson_e	0.632	0.594	0.040

1 *CARN, lambs received a diet including 3 g of L-Carnitine/kg*.

**Figure 1:**
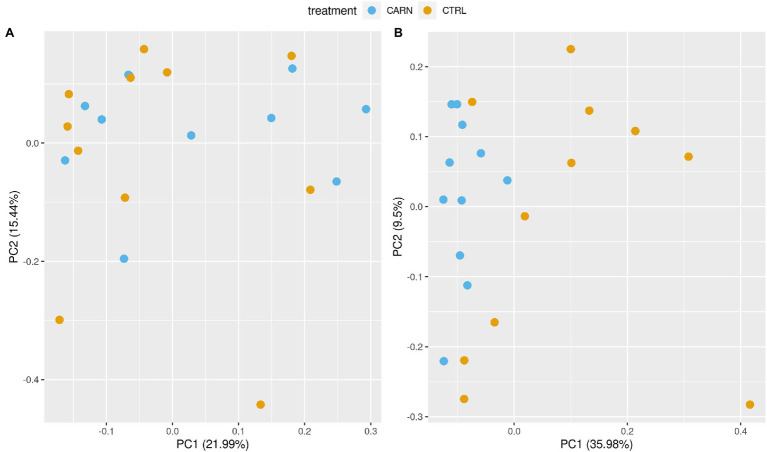
Beta diversity plot. Principal component plots of Bray–Curtis distances between the microbiotas [ileum (left, **A**) and rumen (right, **B**)] of early feed restricted lambs being fed the control (CTRL) or the L-Carnitine diet (CARN) during the fattening period.

Bray–Curtis distances showed clear and statistically significant (value of *p* < 0.001 from PERMANOVA) separation between anatomical compartments (ileum vs. rumen, data not shown). No significant differences were found in Bray–Curtis distances between CARN and CTRL lambs neither in the rumen nor in the ileum (values of *p* from PERMANOVA 0.112 and 0.428, respectively, for rumen and ileum: Figures 1A,B). Nevertheless, some clustering of carnitine and control samples was apparent in the rumen, while no pattern emerged in the ileum.

**Figure 2:**
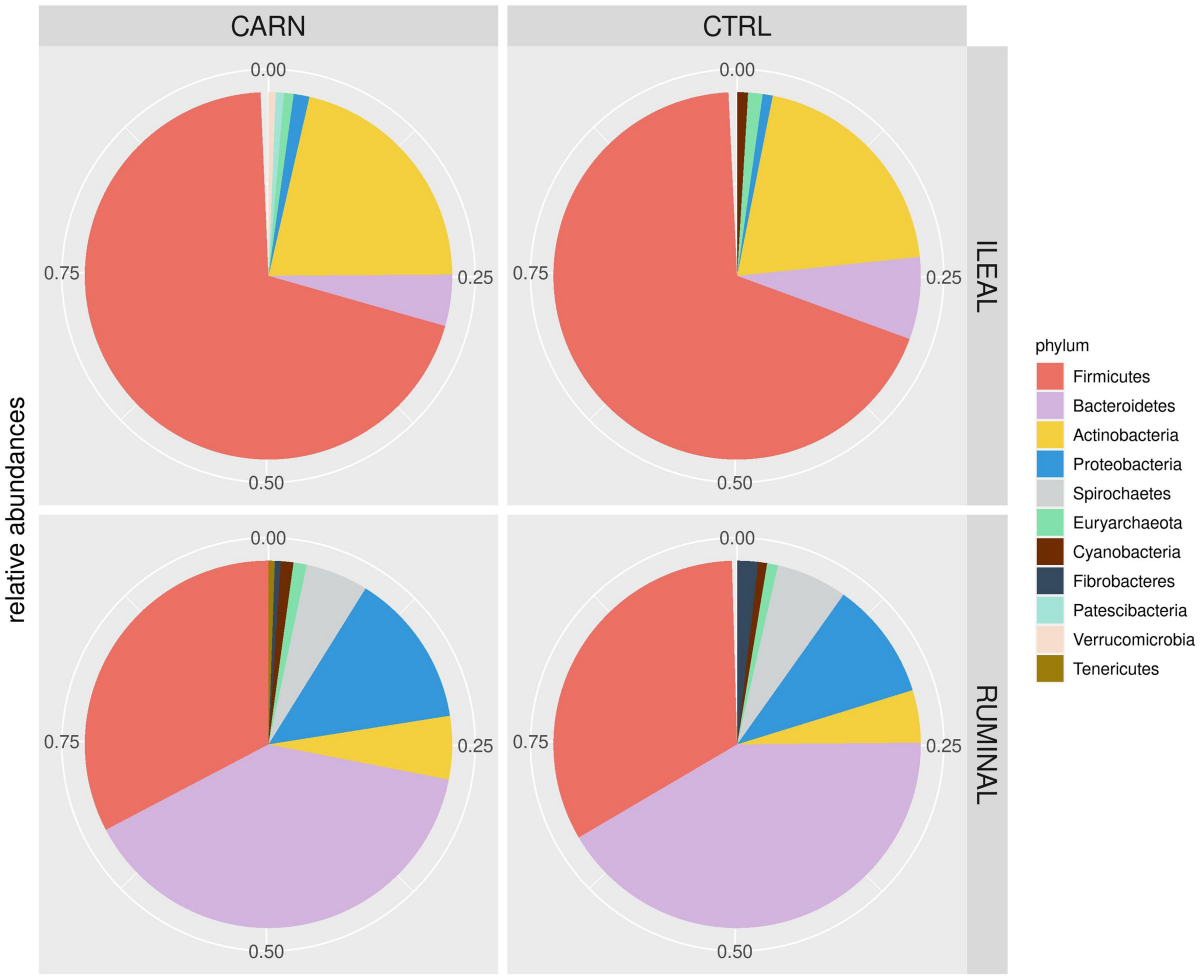
Pie chart of relative abundances at the phylum level in the microbiota of ileum and rumen of early feed restricted lambs being fed the control (CTRL) or the L-Carnitine diet (CARN) during the fattening period.

*Firmicutes* were found to be the most abundant phylum in the ileum microbiota, while the phylum *Bacteroidetes* was predominant in the rumen microbiota (Figure 2). Other major phyla included *Actinobacteria* in the ileum and *Proteobacteria* in the rumen. When looking at the differences between experimental groups, 28 and 12 OTUs were found to be significantly different in carnitine vs. controls in the rumen and ileum, respectively (Table 7), including most of the species that are regarded as part of the ruminal bacterial community, such as *Ruminococcus, Butyrivibrio*, *Lachnospiraceae*, and sulfate-reducing bacteria *Desulfobulbus* and *Desulfovibrio*.

**Table 7:** List of OTUs (ruminal and ileal compartments) with relative abundances significantly different in the early feed restricted lambs being fed the control (CTRL) or the L-Carnitine diet (CARN) during the fattening period.

Level	Taxa	Type	CTRL	CARN^1^	Value of *p*
Class	Anaerolineae	rumen	8.02E-05	3.38E-05	0.022
Family	Anaerolineaceae	rumen	8.02E-05	3.38E-05	0.022
Family	Clostridiales vadinBB60 group	rumen	3.89E-03	2.17E-04	0.010
Family	Desulfobulbaceae	rumen	6.39E-05	2.62E-05	0.017
Genus	[Eubacterium] hallii group	rumen	7.60E-05	4.84E-05	0.047
Genus	Alistipes	rumen	2.05E-04	4.38E-05	0.007
Genus	Butyrivibrio 2	rumen	4.44E-04	1.76E-04	0.044
Genus	Desulfobulbus	rumen	6.39E-05	2.62E-05	0.017
Genus	Erysipelotrichaceae UCG-004	rumen	3.23E-03	8.27E-04	0.026
Genus	Flexilinea	rumen	8.02E-05	3.38E-05	0.022
Genus	Howardella	rumen	1.95E-04	1.10E-04	0.041
Genus	Klebsiella	rumen	0.00E+00	1.09E-05	0.006
Genus	Lachnospiraceae FCS020 group	rumen	5.13E-05	1.74E-05	0.012
Genus	Lachnospiraceae UCG-006	rumen	1.08E-04	7.47E-05	0.033
Genus	Lachnospiraceae UCG-008	rumen	1.34E-03	6.70E-04	0.009
Genus	Lachnospiraceae UCG-010	rumen	3.67E-05	1.04E-05	0.016
Genus	Moryella	rumen	4.09E-05	2.09E-04	0.010
Genus	Oribacterium	rumen	6.31E-04	2.10E-04	0.040
Genus	Prevotellaceae UCG-003	rumen	3.45E-03	1.12E-03	0.037
Genus	Pyramidobacter	rumen	1.52E-03	8.96E-04	0.032
Genus	Ruminococcaceae NK4A214 group	rumen	2.59E-03	1.77E-03	0.031
Genus	Ruminococcaceae UCG-004	rumen	2.34E-04	1.08E-04	0.003
Genus	Ruminococcus 1	rumen	4.72E-03	1.24E-03	0.023
Genus	Slackia	rumen	2.12E-06	1.74E-04	0.000
Genus	Tyzzerella	rumen	4.18E-05	6.66E-06	0.031
Order	Anaerolineales	rumen	8.02E-05	3.38E-05	0.022
Order	Desulfobacterales	rumen	6.39E-05	2.62E-05	0.017
Phylum	Chloroflexi	rumen	8.02E-05	3.38E-05	0.022
Class	Clostridia	ileum	3.36E-01	4.10E-01	0.013
Family	Ruminococcaceae	ileum	1.88E-01	2.78E-01	0.025
Family	Sphingomonadaceae	ileum	1.44E-04	1.10E-05	0.021
Genus	Anaerostipes	ileum	5.45E-06	4.85E-05	0.036
Genus	Romboutsia	ileum	6.38E-03	5.20E-04	0.045
Genus	Ruminiclostridium 5	ileum	1.85E-05	6.78E-05	0.023
Genus	Slackia	ileum	0.00E+00	3.25E-05	0.040
Genus	Sphingomonas	ileum	1.27E-04	1.10E-05	0.025
Order	Clostridiales	ileum	3.36E-01	4.10E-01	0.013
Order	Sphingomonadales	ileum	1.44E-04	1.10E-05	0.021
Phylum	Firmicutes	ileum	6.84E-01	6.97E-01	0.023
Phylum	Lentisphaerae	ileum	3.12E-05	0.00E+00	0.041

1 *CARN, lambs received a diet including 3 g of L-Carnitine/kg*.

### Ileal Morphometrical and Immunological Parameters

Ileal morphometrical and immunological parameters are shown in Table 8. Carnitine supplementation did not affect (*p* > 0.05) the thickness of ileal layers of the crypt depth, but the length of the villi was significantly greater (value of *p* < 0.05) in lambs of the CARN group. There were no differences (*p* > 0.05) between treatments in T infiltrating lymphocyte counts, but B lymphocyte counts trended (*p* = 0.079) to be lower for CARN lambs. M cells, IgA concentration, and the relative RNAm abundance (Table 9) of cytokines and TLRs involved in the innate immune response were not affected (*p* > 0.05) by dietary treatments. Fold change due to carnitine supplementation were −1.10, −1.09, −1.15, −1.13, −1.27, −0.07, −0.99, and −1.01 for IFN, TGF; LI-4, IL-10, TLR3, TLR6, TLR7, and TLR10, respectively.

**Table 8:** Thickness of ileal layers, villus length, crypt depth, infiltrating lymphocyte counts in ileal lamina propria, intensity of staining of M cells in ileal mucosa and ileal IgA concentration of early feed restricted lambs being fed the control (CTRL) or the L-Carnitine diet (CARN) during the fattening period.

	CTRL	CARN^1^	SED^2^	Value of *p*
*Thickness (μm)*				
Mucosa	102	108	6.05	0.331
Submucosa	140	141	9.12	0.930
Muscular	16.6	19.0	1.59	0.134
Villus length (μm)	59.6	70.7	5.83	0.076
Crypt depth (μm)	20.6	21.5	2.20	0.687
*Lymphocytes (number per field 40×)*				
CD3 positive (T cells)	159	138	14.9	0.176
CD20 positive (B cells)	4.21	2.75	0.786	0.079
*Intensity of cytokeratin 18 positive (M cells)*				
High positive	0.058	0.082	0.038	0.543
Medium positive	3.63	4.92	1.08	0.248
Low positive	40.9	48.2	4.46	0.118
Negative	55.4	46.8	5.06	0.104
IgA (pg IgA/μg total protein)	13.1	11.2	1.27	0.146

1 *CARN, lambs received a diet including 3 g of L-Carnitine/kg*.

2 *SED, standard error of the difference*.

**Table 9:** Cytokines and toll-like receptors (TLRs) mRNA expression in the ileal mucosa of early feed restricted lambs being fed the control (CTRL) or the L-Carnitine diet (CARN) during the fattening period.

	CTRL	CARN	SED^6^	Value of *p*
*Cytokines (2^−ΔCt^)^1^*				
IFN-γ^2^	0.00021	0.00019	0.00010	0.808
TGF-β^3^	0.01060	0.00970	0.00106	0.423
IL-4^4^	0.00003	0.00003	0.00001	0.717
IL-10^5^	0.00069	0.00061	0.00012	0.570
*TLR (2^−ΔCt^)*				
TLR_3_	0.0006	0.0004	0.00015	0.436
TLR_6_	0.0094	0.0097	0.00143	0.831
TLR_7_	0.0027	0.0028	0.00080	0.948
TLR_10_	0.0047	0.0045	0.00068	0.841

1 *Ct = quantification cycle. ΔC_t_ = C_t(cytokines or TLRs)_–C_t(β-actin)_. Higher 2^−ΔCt^ values represent higher RNA abundance*.

2 *IFN-γ, interferon gamma*.

3 *TGF-β, transforming growth factor β*.

4 *IL-4, interleukin 4*.

5 *IL-10, interleukin 10*.

6 *SED, standard error of the difference*.

## Discussion

The present study reports the effects of dietary L-Carnitine administration during the fattening period of early feed restricted lambs on biochemical profile, feed efficiency, the ruminal and intestinal microbiome, and intestinal immune system. As a prerequisite of this study, the concentration of plasma carnitine and carnitine derivatives was measured to evaluate the absorption of this compound. Compared with CTRL lambs, administration of dietary carnitine increased carnitine and three acyL-Carnitines concentrations in plasma, thus indicating that some L-Carnitine could escape from intraruminal degradation, being available for absorption in the small intestine. Dietary carnitine in ruminants is partially degraded in the rumen by microorganisms that metabolize carnitine to trimethylaminoacetone, trimethylamine, and Y-butyrobetaine before being excreted in urine or feces (LaCount et al., 1995). Moreover, it is well known that acyL-Carnitines are originated from the intracellular coupling of carnitine to fatty acids. Therefore, the increased concentration of acyL-Carnitines in the blood of the CARN group indicates that, despite ruminal degradation, the supplemented L-Carnitine was absorbed and reached the cytosol of the cells in the fattening lambs. However, in agreement with LaCount et al. (1996b) when supplying doses lower than 7 g/day of L-Carnitine in the diet of lactating dairy cows or with Solhjo et al. (2014), who supplemented fattening lambs with until up to 1.5 g/day of rumen protected L-Carnitine, we observed no differences in animal performance or feed efficiency. Higher doses up to 12 g/day of L-Carnitine when feeding cows did not promote improvements either (LaCount et al., 1996a). Greenwood et al. (2001) neither observed improvements in feed efficiency when adding 2 g/day of L-Carnitine to the diet of growing steers. Accordingly, and considering that supplementation of unprotected carnitine increased systemic carnitine levels, the lack of effect of dietary L-Carnitine on feed efficiency is not provoked by intraruminal degradation (Ringseis et al., 2018). Conversely, ruminal fermentation was improved when feeding L-Carnitine to the lambs, as suggested by the increment of total SCFA in the rumen. LaCount et al. (1995) also detected increments (*p* < 0.09) of total SCFA in the ruminal fluid of cannulated cows when 6 g/day of L-Carnitine were infused directly into the rumen, together with a trend toward increased dry matter digestibility.

Furthermore, in agreement with LaCount et al. (1995), a reduction in cholesterol content was found with dietary L-Carnitine administration, which has been attributed to a decrease of carnitine-induced acetyl coenzyme A (essential in cholesterol synthesis) caused by free carnitine combining with acetyl coenzyme A to form acetylcarnitine (Stephens et al., 2007). Moreover, considering that net lipid mobilization is minimal during the fattening period, it is not surprising that no effects on NEFA and BHB were found. In agreement with our results, Greenwood et al. (2001) also reported the lack of effect of L-Carnitine on lipomobilization in growing steers using lower doses. In the present study, other results, such as reduced numerical values of enzyme markers (e.g., AST) for detecting liver injury, may be indicating that L-Carnitine can protect cell membrane and mitochondria of hepatocytes from damage due either to its antioxidant properties or the ability to increase β-oxidation, thus reducing both the oxidative stress (which induces impairments in mitochondrial β-oxidation) and the accumulation of fatty acids. Nevertheless, the lack of significant differences in both, SOD and triglycerides between groups points toward different reasons behind the reduced numerical values of AST in the CARN group. Accordingly, elevated AST levels are not specific to liver disease, but are also related to skeletal muscle metabolism and cardiomyopathy (Pirmadah et al., 2020). At this point, it must be remembered that early feed restricted lambs showed increased catabolism of proteins and hypertrophic cardiomyopathy caused by mitochondrial dysfunction (Santos et al., 2018b), so dietary L-Carnitine might have exerted some protective effects in the present study; the reduced heart weight in the CARN group together with reduced numerical levels of CK, a marker of muscle metabolism and damage (Yarizadh et al., 2020), would be aligned with this hypothesis. It is also remarkable the reduced serum creatinine levels in the CARN group, another indicator of improved cardiac and renal functions (Abu Ahmad et al., 2016). Regardless of the lack of improvement of feed efficiency traits, all these positive effects achieved in early feed restricted lambs with the administration of dietary L-Carnitine contradict previous statements hold in other studies (Greenwood et al., 2001), where it was suggested that the endogenous carnitine synthesis was sufficient to facilitate carnitine-dependent functions in intermediary metabolism.

Moreover, a systematic review and meta-analysis demonstrated that L-Carnitine had a significant reducing effect on ammonia levels caused by the upregulation of genes involved in the ureagenesis (Abbasnezhad et al., 2019). This seems to agree with our results because even though no effects were observed at ruminal ammonia nitrogen (NH_3_-N) concentration (*p* > 0.05), a trend toward increased concentration in the plasmatic urea values was detected in the CARN group (*p* = 0.099). Therefore, L-Carnitine may have beneficial effects in growing ruminants experiencing hyperammonemia induced by feeding excess sources of non-protein nitrogen. On the contrary, dietary L-Carnitine appears to be largely unsuccessful for improving the performance of growing ruminants being fed a diet with adequate crude protein content, as happened in the present study.

When the effects of L-Carnitine on ruminal microbiota were assessed no clear clustering of samples related to dietary L-Carnitine feeding treatment was observed demonstrating that feeding L-Carnitine does not induce any major shift in the most abundant bacterial population. Still, the treatment with L-Carnitine had an impact on what has been defined as the rumen core microbiota population, causing a statistically significant decrease of bacteria belonging to the *Ruminococcus*, *Butyrivibrio*, and other *Lachnospiraceae* taxa, which are involved, as cellulolytic bacteria, in the breakdown of cellulose and the consequent release of SCFAs, such as acetate, propionate, and butyrate (Anderson et al., 2021). This would also explain the trend toward significant higher values of digestibility observed in the CARN group. Moreover, the increased relative abundance of *Succinivibrionaceae* (associated with starch degradation) in the rumen was also probably related to the higher production of propionate (Iqbal et al., 2018) whereas the decreased proportion of bacteria *Desulfobulbus* (involved in propionate degradation) may have also contributed to increasing the proportion of this SCFA (Kremer and Hansen, 1988). It is also remarkable the increased proportion of *Slackia* in the rumen (also in the ileum), a genus that has been previously related to improved feed efficiency in the chicken (Wen et al., 2021).

Regardless of the effects of L-Carnitine on microbiota composition, it must be taken into account that ruminants fed starch-rich diets accumulate organic acids in the rumen, decreasing both pH and motility and increasing osmotic pressure (Nagaraja and Titgemeyer, 2007; Hernández et al., 2014). Although this effect is counteracted by water flux from the bloodstream (through saliva or directly entering across rumen wall) L-Carnitine, a compound with osmoprotectant properties, may have allowed more susceptible bacteria to overcome osmotic stress (Meadows and Wargo, 2015), whereas it is plausible that microbiota of the control lambs had to use some nutrients to synthesize L-Carnitine or other osmoprotectants to be adapted against changes in osmotic pressure, thus modifying ruminal fermentation parameters. Likewise, it has been reported that carnitine supplementation can modulate gut motility (Meyer et al., 2020) and transit time, which is another factor that affects gut microbiota composition and fermentation (Tottey et al., 2017; Adebayo Arowolo et al., 2022).

Additionally, dietary L-Carnitine might have also modified the metabolism of ruminal bacteria [e.g., increasing glycine production that can be used as carbon, nitrogen, and energy source (Rebouche and Seim, 1998; Wargo and Hogan, 2009; Wargo, 2013; Ringseis et al., 2018)] thus promoting increments of total SCFAs in the CARN group regardless of the modification of relative abundance of the OTUs previously mentioned. In addition, L-Carnitine can be also transformed into trimethylamine, generating malic acid (Kleber, 1997) that enhances ruminal fermentation (and digestibility), increasing both the total concentration of SCFA and the molar proportion of propionic acid (Liu et al., 2009), as happened in the current study. With the independence of the mechanism causing the increased amounts of SCFA in the CARN group, LaCount et al. (1995) also found increased proportions of propionate, reductions in isovalerate, and no changes in the proportions of acetate in the rumen when feeding dietary carnitine to lactating dairy cows. According to Paula et al. (2017), a reduced amount or degradation of branched-chain amino acids may originate a lower molar ratio of both isovalerate and isobutyrate. However, in our study, no differences were observed between treatments when isovalerate and isobutyrate were expressed as mmol/L, so the reduced proportions were probably caused by a dilution effect due to a greater amount of SCFA, basically propionate, driven by a larger carbohydrate fermentation. Nevertheless, data obtained from the digestive content should be interpreted with caution, since lambs were fasted for 2 h before slaughter. In any case, according to these results, carnitine might benefit hepatic gluconeogenesis (van Soest, 1994) because in the post-absorption propionate is converted to glucose in the liver for energy supply. Moreover, even though no changes in the molar proportions of acetate were found in any of these studies, the increments of total SCFA production in the rumen might have provided more substrates (e.g., acetate) for fat syntheses (e.g., intramuscular fat) in the CARN group. In any case, all these mechanisms would explain the higher intramuscular fat content when feeding dietary L-Carnitine (Table 3), an opposite effect to the increased β-oxidation of fatty acids at the mitochondrial level achieved by this compound. Both reverse and simultaneous outcomes would explain, at least partially, the lack of differences in feed efficiency when measured in terms of weight gain (Santos et al., 2018b). Greenwood et al. (2001) also observed (unexpectedly) numerical increments (e.g., carcasses grading USDA, dressing, and intramuscular fat) in some fat depots of growing steers being fed L-dietary carnitine whereas others (e.g., pelvic, kidney, and heart depots) were not modified by L-Carnitine supplementation, so they suggested the existence of specific L-Carnitine effects to certain fat depots.

Changes in the ileal SCFAs profile were found as well, being remarkable the trend toward significant increments in the molar proportions of butyrate in the CARN group, probably explained by microbiome changes at this location. This is important because many *in vitro* studies have shown that SCFAs, and particularly butyrate, may supply energy and stimulate epithelial cell proliferation in the small intestine in a dose-dependent manner (Diao et al., 2019), so nutrient absorption and the integrity of the gut barrier function might be improved. In fact, ileum villus length was greater in CARN lambs and DM digestibility showed a trend toward higher values either. Moreover, previous studies have shown that butyrate at the ileal level seems to reduce the adherent ability of *Escherichia coli*, whereas that of *Lactobacillus acidophilus* and *Bifidobacterium longum* seems to be increased (Jung et al., 2015), which at the end might explain partially the anti-inflammatory properties attributed to this SCFA (Mishiro et al., 2013). None of these species was significantly modified in the present study. However, in agreement with previous studies, increments in the intestinal abundance of *Anaerostipes* were correlated to higher concentrations of plasma butyric acid (Shetty et al., 2020; Bui et al., 2021), which also showed a trend toward significantly higher values in the ileum of the CARN lambs. Moreover, an increase in *Ruminoclostridium*, a cellulosome-producing bacterium being able to metabolize branched plant polysaccharides, such as xyloglucan, was also observed in the CARN group (Ravachol et al., 2016). On the contrary, other bacterial genera that were reported as increasing butyrate concentration in the sheep species, such as *Sphingomonas* (Zhang et al., 2021), were reduced in CARN animals, whereas a trend toward decreased proportions of acetate (another SCFA also related to the regulation of intestinal inflammation and highly produced by *Bifidobacteria* species; Yao et al., 2021) was also detected. In any case, all these effects of dietary L-Carnitine at the ileal level were not enough to cause significant changes in the morphometrical analyses of the ileal samples (Table 8), the immunohistochemistry markers (B and T infiltrating lymphocyte counts in ileal lamina propria and M cells), ileal IgA (Table 8), or in the ileal gene expression of cytokines and toll-like receptors (TLRs) involved in the innate immune response (Table 9).

## Conclusion

According to the results of the present study, the administration of 3 g L-Carnitine/kg CPD during the fattening period of early feed restricted lambs enhances ruminal fermentation, modifies gut microbiota, and improves markers related to muscle damage, renal function, and ureagenesis. However, its effect on feed efficiency during the fattening period of early feed restricted lambs is disappointing, probably because the improved ruminal fermentation promoted a greater accumulation of fat depots, which might have hidden the effects caused by the ability of dietary L-Carnitine to increase fatty acid oxidation at the mitochondrial level. Accordingly, lower doses of dietary L-Carnitine should be tested in early feed restricted lambs, and probably in the context of animals being fed in excess non-protein nitrogen, which should be considered the mainline to conduct future experiments testing the potential of dietary L-Carnitine in livestock.

## Data Availability Statement

The 16S rRNA gene sequences obtained from this study were deposited in the EMBL-EBI European Nucleotide Archive (ENA) repository with the accession number PRJEB49513.

## Ethics Statement

The animal study was reviewed and approved by CSIC Animal Experimentation Committee.

## Author Contributions

AM revised the previous literature and wrote sections of the manuscript and contributed to the in vivo experiment and laboratory analyses. SA and FG contributed to conception and design of the study. PC and BC sequenced the DNA microbiome samples. FB carried out the bioinformatics analyses for microbiome. NS contributed to mRNA extraction and RT-qPCR analyses. FC contributed to discussion of microbiome and structure of the whole paper. FG performed the statistical analysis of several parameters. SA wrote the first draft of the manuscript. All authors contributed to manuscript revision, read, and approved the submitted version.

## Funding

This work was funded by Ministerio de Ciencia e Innovación (RTI2018-099329-B-I00, MCIN/AEI/10.13039/501100011033, “FEDER, Una manera de hacer Europa”).

## Conflict of Interest

The authors declare that the research was conducted in the absence of any commercial or financial relationships that could be construed as a potential conflict of interest.

## Publisher’s Note

All claims expressed in this article are solely those of the authors and do not necessarily represent those of their affiliated organizations, or those of the publisher, the editors and the reviewers. Any product that may be evaluated in this article, or claim that may be made by its manufacturer, is not guaranteed or endorsed by the publisher.

## References

[ref300] Abbasnezhad A. Choghakhori R. Kashkooli S. Alipour M. Asbaghi O. Mohammadi R. (2019). Effect of L-carnitine on liver enzymes and biochemical factors in hepatic encephalopathy: a systematic review and meta-analysis. J. Gastroenterol. Hepatol. 34, 2062–2070. doi: 10.1111/jgh.14765, PMID: 31254469

[ref400] Abu Ahmad N. Armaly Z. Berman S. Jabour A. Aga-Mizrachi S. Mosenego-Ornan E.. (2016). L-Carnitine improves cognitive and renal functions in a rat model of chronic kidney disease. Physiol. Behav. 164, 182–188. doi: 10.1016/j.physbeh.2016.05.036, PMID: 27241631

[ref1] Adebayo Arowolo M. Zhang X. M. Wang M. Wang R. Wen J. N. Hao L. Z.. (2022). Proper motility enhances rumen fermentation and microbial protein synthesis with decreased saturation of dissolved gases in rumen simulation technique. J. Dairy Sci. 105, 231–241. doi: 10.3168/jds.2021-20165, PMID: 34696908

[ref2] Anderson M. J. (2001). A new method for non-parametric multivariate analysis of variance. Austral Ecol. 26, 32–46. doi: 10.1111/J.1442-9993.2001.01070.PP.X

[ref3] Anderson C. J. Koester L. R. Schmitz-Esser S. (2021). Rumen epithelial communities share a core bacterial microbiota: a meta-analysis of 16S rRNA gene illumina MiSeq sequencing datasets. Front. Microbiol. 12:539. doi: 10.3389/FMICB.2021.625400/BIBTEX PMC8005654 33790876

[ref4] Biscarini F. (2017). GitHub. Rare-OTUs-ACE. Available at: https://github.com/filippob/Rare-OTUs-ACE (Accessed October 30, 2021).

[ref5] Biscarini F. Cremonesi P. Castiglioni B. Stella A. Bronzo V. Locatelli C.. (2020). A randomized controlled trial of teat-sealant and antibiotic dry-cow treatments for mastitis prevention shows similar effect on the healthy milk microbiome. Front. Vet. Sci. 7:581. doi: 10.3389/FVETS.2020.00581/FULL, PMID: 32984415 PMC7492605

[ref6] Biscarini F. Palazzo F. Castellani F. Masetti G. Grotta L. Cichelli A.. (2018). Rumen microbiome in dairy calves fed copper and grape-pomace dietary supplementations: composition and predicted functional profile. PLoS One 13:e0205670. doi: 10.1371/JOURNAL.PONE.0205670 30496201 PMC6264861

[ref7] Bray J. R. Curtis J. T. (1957). An ordination of the upland forest communities of southern Wisconsin. Ecol. Monogr. 27, 325–349. doi: 10.2307/1942268

[ref8] Bui T. P. N. Mannerås-Holm L. Puschmann R. Wu H. Troise A. D. Nijsse B.. (2021). Conversion of dietary inositol into propionate and acetate by commensal Anaerostipes associates with host health. Nat. Commun. 12, 4798–4716. doi: 10.1038/s41467-021-25081-w, PMID: 34376656 PMC8355322

[ref9] Cantalapiedra-Hijar G. Abo-Ismail M. Carstens G. E. Guan L. L. Hegarty R. Kenny D. A.. (2018). Review: biological determinants of between-animal variation in feed efficiency of growing beef cattle. Animal 12, S321–S335. doi: 10.1017/S1751731118001489, PMID: 30139392

[ref10] Caporaso J. G. Kuczynski J. Stombaugh J. Bittinger K. Bushman F. D. Costello E. K.. (2010). QIIME allows analysis of high-throughput community sequencing data. Nat. Methods 7, 335–336. doi: 10.1038/NMETH.F.303, PMID: 20383131 PMC3156573

[ref11] Caporaso J. G. Lauber C. L. Walters W. A. Berg-Lyons D. Lozupone C. A. Turnbaugh P. J.. (2011). Global patterns of 16S rRNA diversity at a depth of millions of sequences per sample. Proc. Natl. Acad. Sci. 108, 4516–4522. doi: 10.1073/PNAS.1000080107, PMID: 20534432 PMC3063599

[ref12] Chao A. (1984). Non-parametric estimation of the classes in a population. Scand. J. Stat. 11, 265–270. doi: 10.2307/4615964

[ref13] Chao A. Lee S.-M. (1992). Estimating the number of classes via sample coverage. J. Am. Stat. Assoc. 87, 210–217.

[ref14] Chao A. Yang C. K. Y. (1993). Stopping rules and estimation for recapture debugging with unequal failure rates. Biometrika 80, 193–201.

[ref15] Chavatte-Palmer P. Velazquez M. A. Jammes H. Duranthon V. (2018). Review: epigenetics, developmental programming and nutrition in herbivores. Animal 12, S363–S371. doi: 10.1017/S1751731118001337, PMID: 30139395

[ref16] Diao H. Jiao A. R. Yu B. Mao X. B. Chen D. W. (2019). Gastric infusion of short-chain fatty acids can improve intestinal barrier function in weaned piglets. Genes Nutr. 14:4. doi: 10.1186/s12263-019-0626-x, PMID: 30761185 PMC6359775

[ref17] Fisher R. A. Corbet A. S. Williams C. B. (1943). The relation between the number of species and the number of individuals in a random sample of an animal population. J. Anim. Ecol. 12:42. doi: 10.2307/1411

[ref19] Foundation for Statistical Computing (2015). R: a language and environment for statistical computing. Foundation for statistical Computing. Available at: https://www.gbif.org/es/tool/81287/r-a-language-and-environment-for-statisticaL-Computing (Accessed November 5, 2021).

[ref20] Frutos J. Andrés S. Trevisi E. Benavides J. Santos N. Santos A.. (2018). Moderated milk replacer restriction of ewe lambs alters gut immunity parameters during the pre-weaning period and impairs liver function and animal performance during the replacement phase. Anim. Feed Sci. Technol. 243, 80–89. doi: 10.1016/J.ANIFEEDSCI.2018.07.009

[ref21] Giráldez F. J. Santos N. Santos A. Valdés C. López S. Andrés S. (2021). Fattening lambs with divergent residual feed intakes and weight gains: unravelling mechanisms driving feed efficiency. Anim. Feed Sci. Technol. 273:114821, 114821. doi: 10.1016/J.ANIFEEDSCI.2021.114821

[ref22] Greenwood R. H. Titgemeyer E. C. Stokka G. L. Drouillard J. S. Lö C. A. (2001). Effects of L-carnitine on nitrogen retention and blood metabolites of growing steers and performance of finishing steers. J. Anim. Sci. 79, 254–260. doi: 10.2527/2001.791254x 11204708

[ref23] Hajilou M. Dehghan-Banadaky M. Zali A. Rezayazdi K. (2015). The effects of dietary L-Carnitine and rumenprotected choline on growth performance, carcass characteristics and blood and rumen metabolites of Holstein Young Bulls. J. Appl. Anim. Res. 42, 89–96. doi: 10.1080/09712119.2013.822807

[ref24] Hernández J. Benedito J. L. Castillo C. (2014). Ruminal acidosis in feedlot: from aetiology to prevention. Sci. World J. 2014:702572. doi: 10.1155/2014/702572 PMC4247954 25489604

[ref25] Hill G. M. Newton G. L. Blum S. A. (1995). Carnitine supplementation of feedlot heifer and steer diets. J. Anim. Sci. 73:34.

[ref26] Iqbal M. W. Zhang Q. Yang Y. Zou C. Li L. Liang X.. (2018). Ruminal fermentation and microbial community differently influenced by four typical subtropical forages in vitro. Anim. Nutr. 4, 100–108. doi: 10.1016/j.aninu.2017.10.005, PMID: 30167491 PMC6112341

[ref27] Jonh J. (2011). GitHub. Tool for stripping adaptors and/or merging paired reads with overlap into single reads. Available at: https://github.com/jstjohn/SeqPrep#readme (Accessed October 30, 2021).

[ref28] Jung T. H. Park J. H. Jeon W. M. Han K. S. (2015). Butyrate modulates bacterial adherence on LS174T human colorectal cells by stimulating mucin secretion and MAPK signaling pathway. Nutr. Res. Pract. 9, 343–349. doi: 10.4162/nrp.2015.9.4.343, PMID: 26244071 PMC4523476

[ref29] Kleber H. P. (1997). Bacterial carnitine metabolism. FEMS Microbiol. Lett. 147, 1–9. doi: 10.1111/j.1574-6968.1997.tb10212.x 9037756

[ref30] Kremer D. R. Hansen T. A. (1988). Pathway of propionate degradation in Desulfobulbus propionicus. FEMS Microbiol. Lett. 49, 273–277. doi: 10.1111/J.1574-6968.1988.TB02729.X

[ref31] LaCount D. W. Drackley J. K. Weigel D. J. (1995). Responses of dairy cows during early lactation to ruminal or abomasal administration of L-Carnitine. J. Dairy Sci. 78, 1824–1836. doi: 10.3168/jds.S0022-0302(95)76807-2 8786266

[ref32] LaCount D. W. Emmert L. S. Drackley J. K. (1996a). Dose response of dairy cows to abomasal administration of four amounts of L-Carnitine. J. Dairy Sci. 79, 591–602. doi: 10.3168/jds.S0022-0302(96)76404-4, PMID: 8744224

[ref33] LaCount D. W. Ruppert L. D. Drackley J. K. (1996b). Ruminal degradation and dose response of dairy cows to dietary L-Carnitine. J. Dairy Sci. 79, 260–269. doi: 10.3168/jds.S0022-0302(96)76359-2, PMID: 8708087

[ref34] Lehner R. Quiroga A. D. (2016). “Fatty acid handling in mammalian cells” in Biochemistry of Lipids, Lipoproteins and Membranes (Sixth Edition). eds. Ringway N. D. McLeod R. S. (USA: Elsevier), 149–184.

[ref35] Li W. Godzik A. (2006). Cd-hit: a fast program for clustering and comparing large sets of protein or nucleotide sequences. Bioinformatics 22, 1658–1659. doi: 10.1093/BIOINFORMATICS/BTL158 16731699

[ref36] Liu Q. Wang C. Yang W. Z. Dong Q. Huang Y. X. Yang X. M.. (2009). Effects of malic acid on rumen fermentation, urinary excretion of purine derivatives and feed digestibility in steers. Animal 3, 32–39. doi: 10.1017/S1751731108003364, PMID: 22444170

[ref37] Makovicky P. Tumova E. Volek Z. Makovicky P. Vodicka P. (2014). Histological aspects of the small intestine under variable feed restriction: The effects of short and intense restriction in a growing rabbit model. Exp. Ther. Med. 8, 1623–1627. doi: 10.3892/etm.2014.1924, PMID: 25289070 PMC4186336

[ref38] Martínez-Pérez J. M. Robles-Pérez D. Rojo-Vázquez F. A. Martínez-Valladares M. (2014). Immunological features of LPS from *Ochrobactrum intermedium* on sheep experimentally infected with *Fasciola hepatica*. Res. Vet. Sci. 97, 329–332. doi: 10.1016/j.rvsc.2014.07.015, PMID: 25151434

[ref39] Meadows J. A. Wargo M. J. (2015). Carnitine in bacterial physiology and metabolism. Microbiology 161, 1161–1174. doi: 10.1099/mic.0.000080, PMID: 25787873 PMC4635513

[ref40] Meyer J. Daniels S. U. Grindler S. Tröscher-Mubotter J. Alaedin M. Franhm J.. (2020). Effects of a dietary L-Carnitine supplementation on performance, energy metabolism and recovery from calving in dairy cows. Animals 10:342. doi: 10.3390/ani10020342, PMID: 32098123 PMC7070952

[ref500] Mishiro T. Kusunoki R. Otani A. Ansary M. M. Tongu M. Harashima N.. (2013). Butyric acid attenuates intestinal inflammation in murine DSS-induced colitis model via milk fat globule-EGF factor 8. Lab. Invest. 93, 834–843. doi: 10.1038/labinvest.2013.70, PMID: 23752130

[ref41] Nagaraja T. G. Titgemeyer E. C. (2007). Ruminal acidosis in beef cattle: the current microbiological and nutritional outlook. J. Dairy Sci. 90, E17–E38. doi: 10.3168/jds.2006-478 17517750

[ref600] Paula E. M. Monteiro H. F. Silva L. G. Benedeti P. D. B. Daniel J. L. P. Shenkoru T.. (2017). Effects of replacing soybean meal with canola meal differing in rumen-undegradable protein content on ruminal fermentation and gas production kinetics using 2 in vitro systems. J. Dairy Sci. 100, 5281–5292. doi: 10.3168/jds.2016-12301, PMID: 28456405

[ref42] Paulson J. N. Stine O. C. Bravo H. C. Pop M. (2013). Differential abundance analysis for microbial marker-gene surveys. Nat. Methods 10, 1200–1202. doi: 10.1038/NMETH.2658, PMID: 24076764 PMC4010126

[ref700] Pirmadah F. Ramezani-Jolfaie N. Mohammadi M. Talenezhad N. Clark C. C. T. Salehi-Abargouei A. (2020). Does L-carnitine supplementation affect serum levels of enzymes mainly produced by liver? A systematic review and meta-analysis of randomized controlled clinical trials. Eur. J. Nutr. 59, 1767–1783. doi: 10.1007/s00394-019-02068-4, PMID: 31385062

[ref43] Quast C. Pruesse E. Yilmaz P. Gerken J. Schweer T. Yarza P.. (2013). The SILVA ribosomal RNA gene database project: improved data processing and web-based tools. Nucleic Acids Res. 41, D590–D596. doi: 10.1093/NAR/GKS1219, PMID: 23193283 PMC3531112

[ref800] Ravachol J. de Philip P. Borne R. Mansuelle P. Maté M. J. Perret S.. (2016). Mechanisms involved in xyloglucan catabolism by the cellulosome-producing bacterium Ruminiclostridium cellulolyticum. Sci. Rep. 6:22770. doi: 10.1038/srep22770 26946939 PMC4780118

[ref44] Rebouche C. J. Seim H. (1998). Carnitine metabolism and its regulation in microorganism and mammals. Ann. Rev. Nutr. 18, 39–61. doi: 10.1146/annurev.nutr.18.1.39, PMID: 9706218

[ref45] Ringseis R. Keller J. Eder K. (2018). Regulation of carnitine status in ruminants and efficacy of carnitine supplementation on performance and health aspects of ruminant livestock: a review. Arch. Anim. Nutr. 72, 1–30. doi: 10.1080/1745039X.2017.1421340, PMID: 29313385

[ref46] Santos A. Giráldez F. J. Valdés C. Trevisi E. Lucini L. Frutos J.. (2018a). Milk replacer restriction during early life impairs the live body weight and progesterone patterns of ewe lambs during the replacement period. J. Dairy Sci. 101, 8021–8031. doi: 10.3168/JDS.2018-14648, PMID: 29960776

[ref47] Santos A. Valdés C. Giráldez F. J. López S. France J. Frutos J.. (2018b). Feed efficiency and the liver proteome of fattening lambs are modified by feed restriction during the suckling period. Animal 12, 1838–1846. doi: 10.1017/S1751731118000046, PMID: 29362009

[ref48] Saro C. Mateo J. Andrés S. Mateos I. Ranilla M. J. López S.. (2019). Replacing soybean meal with urea in diets for heavy fattening lambs: effects on growth, metabolic profile and meat quality. Animals 9:974. doi: 10.3390/ani9110974, PMID: 31739618 PMC6912220

[ref900] Schmittgen T. D. Livak K. J. (2008). Analyzing real-time PCR data by the comparative C(T) method. Nat. Protoc. 3, 1101–1108. doi: 10.1038/nprot.2008.73, PMID: 18546601

[ref49] Shannon C. E. (1948). A mathematical theory of communication. Bell Syst. Tech. J. 27, 379–423. doi: 10.1002/J.1538-7305.1948.TB01338.X

[ref50] Shetty S. A. Boeren S. Bui T. P. N. Smidt H. de Vos W. M. (2020). Unravelling lactate-acetate and sugar conversion into butyrate by intestinal Anaerobutyricum and Anaerostipes species by comparative proteogenomics. Environ. Microbiol. 22, 4863–4875. doi: 10.1111/1462-2920.15269, PMID: 33001550 PMC7702098

[ref51] Simon Andrews (2010). A quality control tool for high throughput sequence data. Babraham Bioinformatics. Available at: https://www.bioinformatics.babraham.ac.uk/projects/fastqc/ (Accessed October 30, 2021).

[ref52] Simpson E. H. (1949). Measurement of diversity. Nature 163, 688–688. doi: 10.1038/163688a0

[ref53] Smith B. Wilson J. B. (1996). A consumer’s guide to evenness indices. Oikos 76, 70–82. doi: 10.2307/3545749

[ref54] Solhjoo A. Rowghani E. Bayat A. Zamiri M. J. (2014). The effect of rumen protected L-Carnitine on feedlot performance, carcass characteristics and blood metabolites in Iranian fat-tailed Ghezel lambs. Res. Opin. Anim. Vet. Sci. 4, 192–197.

[ref601] Stephens F. B. Constantin-Teodosiu D. Greenhaff P. L. (2007). New insights concerning the role of carnitine in the regulation of fuel metabolism in skeletal muscle. J. Physiol. 581, 431–444. doi: 10.1113/jphysiol.2006.125799, PMID: 17331998 PMC2075186

[ref55] Tottey W. Feria-Gervasio D. Gaci N. Laillet B. Pujos E. Martin J. F.. (2017). Colonic transit time is a driven force of the gut microbiota composition and metabolism: in vitro evidence. J. Neurogastroenterology Motil. 23, 124–134. doi: 10.5056/jnm16042, PMID: 27530163 PMC5216643

[ref56] Van Keulen J. Young B. A. (1977). Evaluation of acid-insoluble ash as a natural marker in ruminant digestibility studies. J. Anim. Sci. 44, 282–287. doi: 10.2527/jas1977.442282x

[ref57] van Soest P. J. (1994). Nutritional Ecology of the Ruminant. 2nd *Edn*. New York: Cornell University Press.

[ref58] Wargo M. J. (2013). Homeostasis and catabolism of choline and glycine betaine: lessons from Pseudomonas aeruginosa. Appl. Environ. Microbiol. 79, 2112–2120. doi: 10.1128/AEM.03565-12/ASSET/E182D086-36B4-4810-96EC-A609743D4253/ASSETS/GRAPHIC/ZAM9991042600002.JPEG, PMID: 23354714 PMC3623244

[ref59] Wargo M. J. Hogan D. A. (2009). Identification os genes required for Pseudomonas aeruginosa carnitine catabolism. Microbiology 155, 2411–2419. doi: 10.1099/mic.0.028787-0, PMID: 19406895 PMC2857723

[ref60] Wen C. Yan W. Mai C. Duan Z. Zheng J. Sun C.. (2021). Joint contributions of the gut microbiota and host genetics to feed efficiency in chickens. Microbiome 9:126. doi: 10.1186/S40168-021-01040-X, PMID: 34074340 PMC8171024

[ref604] Wheatherburn M. (1967). Phenol-hypochlorite reaction for determination of ammonia. Anal. Chem. 39, 971–974. doi: 10.1021/ac60252a045, PMID: 24579848

[ref61] White T. Fernandez J. Gentry G. Gentry L. DeRouen P. Froetschel M. (2001). Influence of urea alone or combined with fish solubles, fish meal, or feather meal in liquid supplement with and without L-Carnitine on performance and ruminal and m metabolic parameters of weanling calves. Prof. Anim. Sci. 17, 145–153. doi: 10.15232/S1080-7446(15)31615-6

[ref62] White T. W. Fernandez J. M. Harding G. D. Williams C. C. Bateman H. G. Bidner T. D.. (2002). Influence of L-Carnitine on performance and ruminal and blood metabolites of grazing calves and finishing lambs. Prof. Anim. Sci. 18, 59–65. doi: 10.15232/S1080-7446(15)31485-6

[ref63] Wickham H. (2009). ggplot2: Elegant Graphics for Data Analysis. Springer New York.

[ref64] Wilson F. D. Cummings T. S. Barbosa M. M. Williams C. J. Gerard P. D. Peebles E. D. (2018). Comparison of two methods for determination of intestinal villus to crypt ratios and documentation of early age-associated ratio changes in broiler chickens. Poult. Sci. 97, 1757–1761. doi: 10.3382/ps/pex349, PMID: 29351670

[ref602] Yarizadh H. Shab-Bidar S. Zamani B. Vanani A. N. Baharlooi H. Djafarian K. (2020). The effect of L-carnitine supplementation on exercise-induced muscle damage: a systematic review and meta-analysis of randomized clinical trials. J. Am. Coll. Nutr. 39, 457–468. doi: 10.1080/07315724.2019.1661804 32154768

[ref603] Yao S. Zhao Z. Wang W. Liu X. (2021). Bifidobacterium longum: protection against inflammatory bowel disease. J. Immunol. Res. 23:8030297. doi: 10.1155/2021/8030297 PMC8324359 34337079

[ref65] Yilmaz P. Parfrey L. Yarza P. Gerken J. Pruesse E. Quast C.. (2014). The SILVA and “all-species living tree project (LTP)” taxonomic frameworks. Nucleic Acids Res. 42, D643–D648. doi: 10.1093/NAR/GKT1209, PMID: 24293649 PMC3965112

[ref66] Zhang R. Zhong Z. Ma H. Lin L. Xie F. Mao S.. (2021). Mucosal microbiota and metabolome in the ileum of Hu Sheep offered a low-grain, pelleted or non-pelleted high-grain diet. Front. Microbiol. 12:718884. doi: 10.3389/fmicb.2021.718884, PMID: 34512596 PMC8427290

